# Typology of organizational innovation components: building blocks to improve access to primary healthcare for vulnerable populations

**DOI:** 10.1186/s12939-020-01263-8

**Published:** 2020-10-06

**Authors:** Mélanie Ann Smithman, Sarah Descôteaux, Émilie Dionne, Lauralie Richard, Mylaine Breton, Vladimir Khanassov, Jeannie L. Haggerty, On behalf of the IMPACT research team

**Affiliations:** 1 Centre de recherche Charles-Le Moyne - Saguenay-Lac-Saint-Jean sur les innovations en santé, Université de Sherbrooke, Longueuil, Québec, Canada; 2 St. Mary’s Research Centre, McGill University, Montreal, Quebec, Canada; 3 Department of General Practice and Rural Health, Dunedin School of Medicine, University of Otago, Dunedin, New Zealand; 4 Department of Community Health, Université de Sherbrooke, Longueuil, Quebec, Canada; 5 Department of Family Medicine, McGill University, Montreal, Quebec, Canada

**Keywords:** Primary healthcare, Vulnerable population, Health services accessibility, Multiple classification analyses, Organizational innovation

## Abstract

**Background:**

Achieving equity of access to primary healthcare requires organizations to implement innovations tailored to the specific needs and abilities of vulnerable populations. However, designing pro-vulnerable innovations is challenging without knowledge of the range of possible innovations tailored to vulnerable populations’ needs. To better support decision-makers, we aimed to develop a typology of pro-vulnerable organizational innovation components *–* akin to “building blocks” that could be combined in different ways into new complex innovations or added to existing organizational processes to improve access to primary healthcare.

**Methods:**

To develop the typology, we used data from a previously conducted a) scoping review (2000–2014, searched Medline, Embase, CINAHL, citation tracking, *n* = 90 articles selected), and b) environmental scan (2014, online survey via social networks, *n* = 240 innovations). We conducted a typological analysis of the data. Our initial typology yielded 48 components, classified according to accessibility dimensions from the *Patient-Centred Accessibility Framework*. The initial typology was then field-tested for relevance and usability by health system stakeholders and refined from 2014 to 2018 (e.g., combined similar components, excluded non-organizational components).

**Results:**

The selected articles (*n* = 90 studies) and survey responses (*n* = 240 innovations) were mostly from the USA, Canada, Australia and the UK. Innovations targeted populations with various vulnerabilities (e.g., low income, chronic illness, Indigenous, homeless, migrants, refugees, ethnic minorities, uninsured, marginalized groups, mental illness, etc.). Our final typology had 18 components of organizational innovations, which principally addressed Availability & Accommodation (7/18), Approachability (6/18), and Acceptability (3/18). Components included *navigation & information*, *community health worker*, *one-stop-shop*, *case management*, *group visits*, *defraying costs, primary healthcare brokerage*, etc.

**Conclusions:**

This typology offers a comprehensive menu of potential components that can help inform the design of pro-vulnerable organizational innovations. Component classification according to the accessibility dimensions of the *Patient-Centred Accessibility Framework* is useful to help target access needs. Components can be combined into complex innovations or added to existing organizational processes to meet the access needs of vulnerable populations in specific contexts.

## Background

Equity of access to healthcare – and specifically, to primary healthcare – is a core value in public policy and a feature of highly-performing health systems in many high-income countries [1–3]. Although reforms to strengthen primary healthcare often invoke equity as a principal goal, they rarely succeed in adequately reaching vulnerable populations whose needs tend to be more complex than those of the general population [4–9]. Indeed, many innovations to improve access to care tend to favour the wealthiest or most educated segments of the population – as famously captured by the *Inverse Care Law* [10]. Achieving healthcare equity, therefore, requires organizations to implement pro-vulnerable innovations, tailored to reach and meet vulnerable populations’ specific needs [11].

**Fig. 1:**
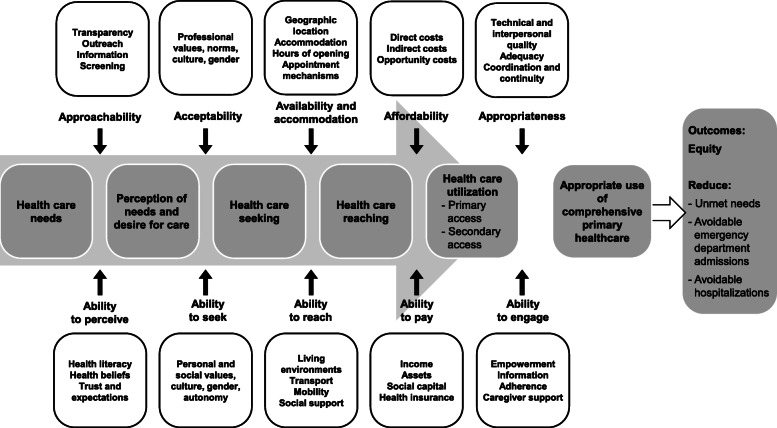
Patient-Centred Accessibility Framework [18]

Vulnerable populations are groups or individuals who are more susceptible to harm because they lack the personal, material, and social resources to successfully cope with the challenges they face and to counter potential harm [12]. These populations are at higher risk for poor health status and problematic access to healthcare [4, 13]. Vulnerable populations include: Aboriginal or Indigenous communities, refugees, visible ethnic minorities, individuals living in poverty, people experiencing homelessness, people with disabilities, people with limited social support, those with complex health conditions, certain age groups; and many other marginalized and underserved populations [4, 13–17]. Inequitable access to healthcare stems from gaps between vulnerable populations’ abilities to access care, and healthcare organizations’ accessibility (see Fig. 1) [18]. To close these gaps, healthcare organizations must adapt their accessibility to vulnerable populations’ abilities. However, designing pro-vulnerable innovations is challenging for decision-makers, providers, and other health system stakeholders, as they may lack knowledge of the range of possible innovations to address vulnerable populations’ specific needs.

This challenge of designing pro-vulnerable innovations was highlighted in our “Innovative Models Promoting Access-to-Care Transformation” (IMPACT) research program [19]. IMPACT brought together local health system decision-makers, providers, primary healthcare researchers, community members, and other stakeholders in six regions in Canada and Australia. In each region, a local multi-stakeholder partnership designed and piloted an organizational innovation aiming to improve access to primary healthcare for vulnerable populations. A foundational premise of the IMPACT program was that innovations should aim to adapt primary care organizations and service delivery arrangements to meet vulnerable populations’ needs, rather than placing the onus solely on vulnerable populations to improve their abilities to access care. To inspire local health system partners, the research team was to provide a menu of existing pro-vulnerable organizational innovations to improve access to primary care.

Substantial research has been conducted on interventions to improve the delivery of care – most notably the taxonomy produced by the Effective Practice and Organisation of Care (EPOC) Group Cochrane Review Group [20]. However, they provide an overview of general interventions that, for the reasons highlighted above, may not address the specific needs of vulnerable populations. Early in the IMPACT program, members of the team conducted a scoping review of studies that described organizational innovations that improved vulnerable populations’ access to primary healthcare – significantly reducing unmet need for care, use of hospital emergency rooms or hospital admissions [17]. This scoping review [17] presented complex interventions, mapped onto the EPOC taxonomy, in which various components of interventions appeared across different interventions. While decision-makers appreciated having results specific to vulnerable populations, they found transposing the results to their contexts challenging and, therefore, of limited use to inform the design of organizational pro-vulnerable innovations.

To better support decision-makers, we aimed to develop a typology of pro-vulnerable organizational innovation *components –* akin to “building blocks” that could be combined in different ways into new complex innovations or added to existing organizational processes. This typology is intended to provide a comprehensive range of components of organizational innovations to be considered by health service decision-makers as options to address the primary healthcare access needs of vulnerable populations.

## Methods

### Design: typology

We developed a typology of components of organizational innovations. A typology is a description and categorization of complex organizational forms [21] developed using qualitative (or quantitative) analysis [22]. The goal of a typology is to divide a whole phenomenon – in our case, organizational innovations to improve access to primary healthcare for vulnerable populations – into distinct but related categories [22, 23]. Typologies have been used in primary healthcare with the intent of guiding organizational change and can provide a “menu” of items to inform the design of interventions [24–29]. In a “typology” – as opposed to a “taxonomy” – items are not ordered hierarchically and are not entirely mutually exclusive.

A qualitative typology is generally structured around a conceptual framework that helps classify emerging categories [22, 23]. In the IMPACT research program, access to primary healthcare for vulnerable populations was conceptualized primarily based on *the Patient-Centred Accessibility Framework* [18]. This framework posits that access to healthcare results from the interaction, at different stages, between organizational dimensions of healthcare and patients’ abilities (Fig. 1). We focused on the dimensions of accessibility on the organizational side of the framework (i.e., Approachability, Acceptability, Availability & Accommodation, Affordability, and Appropriateness).

Inspired by Greenhalgh et al. [30], we defined organizational innovations as: “a novel set of organizational behaviours, routines, and ways of working that are directed at [a common objective] and that are implemented by planned and coordinated actions.” Organizational innovations to improve access to primary healthcare for vulnerable populations were identified from two complementary sources of data: a) a scoping review of the peer-reviewed literature [17] and b) an environmental scan [16]. Both are described briefly below and have been described in detail elsewhere [16, 17]. The scoping review and scan were conducted as part of the IMPACT research program [19].

### Data source: a) scoping review

A scoping review was conducted to explore the breadth of available evidence on organizational innovations in primary healthcare [17]. The search focused on academic, peer-reviewed literature and was conducted in three of the largest and most relevant databases for studies related to primary healthcare (Medline, Embase, and CINAHL). The search was performed by a specialized librarian (see an example of the search strategy in Additional file 1). In addition to the database searches, four primary care experts from the IMPACT team (including JH) were asked to share their personal primary care reference files, from which citation tracking was performed to identify additional relevant studies. The search was limited to articles published between January 2000 and March 2014, a period corresponding to an international commitment to strengthening primary healthcare, up to the beginning of the IMPACT program. One researcher (VK) scanned 8694 titles and abstracts for relevance, then assessed 1760 potentially relevant studies for eligibility. For the typology, we selected any quantitative, qualitative, or mixed methods studies carried out in Organization for Economic Cooperation and Development (OECD) countries and published in English or French that met all four of the following eligibility criteria:

1. involved at least one organization at the primary healthcare level in the health system;
2. was organizational (not directed at the system as a whole or only to the population);
3. had an explicit objective to improve access to care;
4. was directed to a vulnerable population.

The 129 eligible full texts were read by three team members (MAS, JH, SD) to select 90 articles where innovations were described in detail.

### Data source: b) environmental scan

The environmental scan was conducted after the review. It was designed to capture organizational innovations that had not been published in the academic literature [16]. Briefly, a 5-min online survey was disseminated using a social network approach over 6 weeks between July 10 and August 21, 2014. Primary healthcare informants known to the research team were sent a link to the online survey by email and, in turn, were asked to share the survey link within their social networks. The survey was also promoted on social media through 248 posts on Twitter linked to findings of interest and emerging findings from the survey. Participants were invited to identify a program, service, approach, or model of care that they considered innovative in helping vulnerable populations access primary healthcare. They were encouraged to provide links to any available description, such as websites or documents. The definition of an organizational innovation was left to the discretion of the respondents to ease the response burden. We received 744 survey responses. After screening innovations for eligibility and redundancy, 240 unique innovations were retained for the typology.

### Typological analysis

We conducted a typological analysis [22, 23]: first, a) of the selected peer-reviewed articles (*n* = 90) and, subsequently, b) of the selected survey responses (*n* = 240). Each article (n = 90) was carefully read independently by two individuals (MAS, SD), who focused on the ‘types’ of organizational innovations that addressed accessibility and highlighting all passages describing the innovation. The highlighted passages were used to draft a detailed description of each innovation in an Excel document. For each article, we also extracted information about the setting, target population, and vulnerabilities addressed (e.g., frail elderly, homeless, Aboriginal, low income). Reading the detailed description of each innovation, we used a predominantly inductive approach, grouping similar components recurring across organizational innovations. The unit of analysis was a distinct component that could either be a stand-alone intervention (navigation & information), or a combination of components consistently occurring together (e.g., case management, advanced access). We applied standard labels to components where possible (e.g., community health worker) and descriptive labels to others (e.g., proactive identification of need, cultural adaptation).

Subsequently, the emerging typology components were tested by coding the survey responses (*n* = 240) from the scan. Two individuals (MAS, LR) independently coded the responses in an Excel document, then met to resolve discrepancies and discuss possible additions and clarifications to the typology components.

Our initial analysis yielded 48 unique innovation components. Most of the innovations reported in the studies were complex interventions that involved 1–14 components, with an average of six components each. Almost all components were established in the first 40 published studies; analysis of additional published studies and the environmental scan led to refinements. The scoping review and scan data coding were reviewed based on this initial typology and adjusted for consistency and to ensure all relevant components had been captured. We then reviewed the innovations coded to each component of the typology to write a short general description of the component and to select illustrative examples of the component.

Two individuals (MAS, JH) then reviewed the initial typology components, descriptions, and examples and mapped them to the organizational dimensions of the *Patient-Centred Accessibility Framework* [18]. The classifications were based on principal and secondary dimensions of accessibility addressed by the component. Discrepancies in classification were resolved through discussion and checked by the rest of the team.

### Typology field-testing and refinement

The scoping review and environmental scan were conducted in 2014. From 2014 to 2018, the initial typology was presented to academic audiences and field-tested with local partners (e.g., decision-makers, patients, health professionals, researchers) to help them design pro-vulnerable organizational innovations to improve access to primary healthcare. When these initial components were presented to local partners and peers at conferences, they affirmed that the initial 48 components were useful for expanding the options to be considered when designing an innovation. However, they also perceived redundancy between components or lack of direct relevance to accessibility. Therefore, we excluded components that were not organizational components per se*,* but rather resourcing mechanisms: student health professionals, financial incentives, organizational networks, and provider education. We also excluded components that are, in fact, care attributes, such as transparency, advocacy, patient-centred care, and empowerment. Finally, we excluded a few components that applied more to the content or quality of care, such as quality improvement initiatives or self-management education. Components of a similar nature were further collapsed into 18 components of organizational innovations.

## Results

### Organizational innovation characteristics

The 90 selected peer-reviewed studies were set in: the USA (*n* = 60) [31–90], Canada (*n* = 9) [91–99], Australia (*n* = 8) [100–107], UK (*n* = 6) [108–113], New Zealand (*n* = 2) [114, 115], Israel (*n* = 2) [116, 117], Italy (*n* = 1) [118], Mexico (*n* = 1) [119], and Germany (*n* = 1) [120]. For the environmental scan survey, 45.0% of responses originated from Canada, 40.8% from Australia, 9.4% from others countries (e.g., Ireland, UK, USA, Netherlands, Italy, Israel, Switzerland, Cameroon, India, Indonesia, Sudan) and 4.8% were missing country information [16].

The organizational innovations identified in our data targeted a wide variety of vulnerable populations. Targeted populations typically combined various vulnerabilities, most commonly: low-income, chronic illnesses, Indigenous populations, homeless, migrant or refugee status, ethnic minorities, uninsured or underinsured, marginalized groups (drug users, recently incarcerated), persons with mental illness, the frail elderly, at risk youth, and frequent users of emergency departments.

### Typology

The final typology was comprised of 18 components of organizational innovations, presented with examples [42–44, 54–61, 78–83, 94–96, 105, 110, 113, 119–136] in Table 1. The innovation components are organized by the principal accessibility dimension they address. The 18 components principally addressed the dimensions of Availability & Accommodation (7/18), Approachability (6/18), and Acceptability (3/18). Only one component addressed each of Affordability and Appropriateness as principal dimensions, although these were addressed as secondary dimensions as part of other innovation components.

**Table 1:** Typology of organizational innovation components to improve access to primary healthcare for vulnerable populations^a^

Component	Description	Examples	Secondary accessibility dimension
**Principal accessibility dimension: Approachability**			
***Organizational mechanism to make it easier for people facing healthcare needs to identify the available services and how they can be reached***			
**1.** **Proactive identification of need**	A mechanism is put in place to proactively identify vulnerable patients’ need for primary healthcare and provide additional support to avoid the negative consequences of unmet needs.	**Identifying unmet primary healthcare needs in the emergency department:** Patients over the age of 65 with two or more emergency department visits in the previous year not currently in contact with primary care or community services are assigned an advanced practice nurse. The nurse carries out an assessment and physical examination. Case management and referrals are provided as needed [110]. **Regular perinatal home-visits for young mothers:** Women under the age of 19 or under 24 and experiencing social/financial issues and pregnant with their first child enroll in a free public health program. A nurse visits them at home regularly during their pregnancy and up to two years after the child is born. Through proactive follow-ups, the nurse regularly assesses women’s needs, helps access needed services and supports healthy pregnancy, preparation for childbirth, nutrition, exercise, parenting, child development and future life planning [126]. **Canvassing a disadvantaged community for adults living with physical disabilities:** To identify adults living with physical disabilities with unmet long-term care needs at risk of entering nursing homes, community health workers go door-to-door, hand out program literature at community events and accept informal referrals from family, friends and church leaders. Identified patients are then assessed and connected to appropriate community-based resources [60].	Appropriateness
**2.** **Navigation & information**	A service that provides patients with information and support on where, when and how to access primary healthcare.	**Health leads to support low-income patients:** Trained volunteer undergraduate students are available at help desks in medical homes to assist families with social needs connect with community-based resources (e.g., food, housing) and provide support through in-person meetings and telephone follow-ups [42]. **Web-based information system for homeless youth and young adults:** *Healthshack* has online portals including youth-approved community resources and personal records (health, education, housing and employment, a scanned copy of important documents, contact information). Health information includes medical diagnoses, health conditions, prescriptions for medication, laboratory results and referrals to specialists. Public health nurses are available at a youth shelter to help with orientation on the portal, to enter medical history, to provide health counselling and education, to evaluate acute and chronic conditions and to refer youth to appropriate services [58].	Appropriateness
**3.** **Primary healthcare service brokerage**	A service that helps connect vulnerable patients to a primary care provider or primary healthcare service, including single entry points to access with priority queuing by vulnerability indicators.	**Enrollment with a primary care provider for uninsured patients visiting the emergency department:** Health promotion advocates assist uninsured patients visiting the emergency department without a primary care provider to find one. If patients agree, their information is faxed to a caseworker in the chosen primary care site, who then contacts patients to schedule an appointment [43]. **A single-entry model for frail older people with complex needs:** A range of services to maintain the autonomy of older people are available through a single entry point that can be accessed through a telephone line or written referral. Patients are then connected to all needed services (e.g., home care, rehabilitation, community action, case management) [96]. **Centralized waiting list and transitional clinic for patients with chronic disease:** A registered nurse assesses patients who are registered on a centralized waiting list waiting to be connected to a regular primary care provider. Patients with chronic diseases (e.g., diabetes) are prioritized on the waiting list and referred to a transition clinic that provides primary care until patients find a regular provider [125].	
**4. Outreach of primary healthcare services**	Extension of primary healthcare services beyond the physical limits of primary care settings to reach vulnerable populations.	**Mobile health bus for patients experiencing homelessness:** The *Alex Health Bus* (community health bus, dental health bus and youth health bus) provides direct services, advocacy and education in various locations to reach vulnerable patients living in poverty and experiencing homelessness. Services include full checkups, mental health assessments, follow-up care, health promotion, pregnancy testing, sexually transmitted infection testing and treatment, birth control, lab equipment for cholesterol, glucose and urine tests, and referrals to specialists and community resources [134]. **Screening at parish food banks in low-income neighbourhoods:** Parish nurses and a pharmacy school collaborate to offer preventive services (e.g., screening for blood pressure, bone density, cholesterol, glucose, body mass index) out of a mobile health van during regular monthly visits to food banks in parish churches [79].	Availability and accommodation
**5. Inter-sectoral/organizational care pathways**	Primary healthcare organizations collaborate with other organizations (within or outside the health system) to establish procedures that facilitate timely access to needed services for vulnerable groups.	**Early response team for mental health crisis:** *Policy, Ambulance and Clinical Early Response (PACER)* is a joint crisis response unit composed of a police officer and a mental health provider. *PACER* can be called by ambulances or community police when a person is experiencing a mental health crisis. The unit then provides clinical assessment and advice on appropriate transportation options, de-escalation tactics, intervention strategies and referral options for additional services [123, 124]. **Multisectoral space for indigenous women:** The *Casa de la Mujer Indigena* – established through collaboration between non-governmental organizations, local indigenous community members and public institutions – delivers health education and basic healthcare to indigenous women. Indigenous community health workers deliver the services. The space serves as a link to mainstream health services and provides a facilitated referral mechanism for reproductive healthcare. The space also allows community health workers to meet with traditional birthing attendants, medical professionals, non-profit organizations working in reproductive rights and domestic violence to develop services provided to the women of the community [119].	Availability and accommodation
**6. Proactive appointment-making and proactive contact**	Appointment-making processes in primary healthcare that pull-in vulnerable patients to care and maintain ongoing contact.	**Integrated mental health service**: A multidisciplinary health team providing integrated mental health services in a primary care setting, use personalized telephone reminders for appointments and schedule the next appointment immediately after consultation to reduce barriers to attendance. In the event of a missed appointment, staff proactively follow-up by contacting the patient, their family members or other professionals [127]. **Harm reduction and human immunodeficiency virus (HIV)/acquired immune deficiency syndrome (AIDS) primary care:** HIV/AIDS primary care services are integrated into a harm reduction program already offering services to the target population (syringe exchange, housing, job readiness, support groups). Harm reduction outreach teams help participants immediately access primary care, assist them with making appointments, accompany participants to the clinic, help fill out paperwork at the clinic and provide opportunities for reengagement in primary care after a missed appointment [61].	Availability and accommodation
**Principal accessibility dimension:** **Acceptability**			
***Adaptation of services to enhance the social and cultural factors that determine the possibility for people to accept the aspects of a service.***			
**7. Culturally adapted services**	Adapting primary healthcare to the needs of a specific vulnerable group by addressing linguistic or cultural barriers.	**Insurance support for uninsured Latinos:** The *Latino Health Insurance Program* recruits community leaders that reflect the countries of origin of the residents to be case managers. The program provides all communications in English and Spanish and reaches out to the Latino community by deploying case managers to public housing, bodegas, beauty salons, churches, laundromats, etc. Case managers hold educational sessions to help fill out insurance forms in trusted community locations where they provide child care and ethnically-appropriate food. They follow-up with families in a local church office to help maintain insurance coverage and facilitate access to other needed services (e.g., legal services, food stamps) [55]. **Indigenous health center**: The *Inala Indigenous Health Service* consulted with members of the Inala community to identify barriers and facilitators to accessing care. Indigenous staff, including an aboriginal doctor, nurse and health worker, follow ongoing cultural awareness training. Culturally-adapted health posters and Aboriginal and Torres Strait Islander artifacts are displayed in the waiting room, and an aboriginal radio station is played. The service collaborates with community Elders to build ties to the community. It holds various community-based health activities at the Elders’ building (e.g., rugby league, chronic disease self-management, child playgroups). A range of services are provided in a one-stop-shop approach (e.g., mental health, alcohol and other drug services, child health services, retinal photography) [105]. **Pharmacy translation software for non-English speakers:** A software created by a social enterprise is used by pharmacists to accurately translate and print bilingual pharmacy labels and medicine summary sheets for ethnic minority patients that have a limited proficiency in English [129].	
**8. Community health worker**	A layperson–a trusted member of a community or with a close understanding of it–acts as a frontline worker who helps bridge cultural and linguistic barriers for members of the community and facilitates their access to primary healthcare.	**Health promotion by lay community members for disadvantaged families:** *GO-Healthy* aims to promote health, medical examinations and immunization among children from disadvantaged families. Lay women living in the target district who are already involved in community associations – particularly those from migrant or low socioeconomic backgrounds – are trained in health education to lead activities for mothers. Also, personnel from barbershops, nail studios and food stores are recruited and trained to inform and encourage mothers to get a medical examination for their children [120]. **Healthcare navigation by community health workers for Hispanic community members:** *La Vida* trains Hispanic community members to act as *Promotores* to help hard-to-reach Hispanics who have or are at risk for diabetes access social and health services. *Promotores* enroll participants in educational and physical activities, support lifestyle changes, help participants enroll with appropriate insurance programs and provide family support [80].	
**9. Group visits**	Primary healthcare provided to a group with similar vulnerabilities or conditions rather than on an individual basis.	**Group medical visits for low-income minorities:** In community health centers serving low-income minorities, women ages 40–64 with at least one chronic disease who had six or more clinic visits in the previous year are invited to participate in six 90-min visits over nine months. Visits are facilitated by nurse practitioners and physicians and are intended to replace visits to patients’ primary care providers. Each visit consists of a check-in (review action plan, physical examination, discussion), group learning on a specific topic (e.g., exercise, healthy eating), a brief one-on-one encounter to discuss individual treatment plans (discussed in the group), a question period and optional private examinations [81].	Appropriateness
**Principal accessibility dimension: Availability & Accommodation**			
***The organizational mechanisms that make services available and reduce the space, time and process barriers for the services to be reached and used in a timely manner by a wide range of persons.***			
**10. Expanded hours**	A primary healthcare organization expands its opening hours beyond 9-to-5 business hours to accommodate the needs of vulnerable populations.	**Mobile clinics offering primary care in the evening:** Services are offered in the evening or on weekends to accommodate the schedules of marginalized populations such as migrant populations, asylum seekers and the homeless [59, 133]. **Expanded hours and 24/7 telephone triage for uninsured patients:** A managed care plan (enrollment with a primary care provider, case management, co-location of social and mental health services, reduced medication costs, etc.) is offered to uninsured patients below the poverty line, including a large proportion of working poor. Primary care services have expanded hours to cover evenings and provide a 24-h telephone triage service, so patients do not have to miss work to access care [44].	
**11. Advanced access**	A scheduling system that provides urgent care by a known primary healthcare team, triggers planned appointments where needed and allows patients to schedule an appointment at the appropriate time.	**Advanced access for patients with depression:** Primary care settings offer advanced access (also known as open or same-day access) to mental health services for patients suffering from depression. This system is intended to allow patients to access care when they need it and feel ready to engage [83, 113].	Appropriateness
**12. Virtual health services**	Use of videoconferencing, phone, email, text message, apps, etc. for consultations or for monitoring health conditions.	**Telehomecare to support self-monitoring in elderly patients:** Elderly patients suffering from severe chronic conditions (e.g., chronic obstructive pulmonary disorder, cardiac insufficiency) with frequent emergency visits or hospitalizations are provided with monitoring equipment at home (e.g., scale, thermometer, sphygmomanometer, oximeter, pulse). Patients are responsible for taking and sending required measures daily to a nurse in a primary care organization, who responds to alerts and follows-up with patients over the phone or during a home visit [94]. **Telehealth expertise to support rural primary care providers caring for complex patients:** Primary care providers in rural communities or remote settings use *ECHO*–a telehealth technology–to co-manage their patients with Hepatitis C and discuss best practices and treatment options with a “knowledge network” of interdisciplinary specialists (e.g., psychiatry, infectious disease, gastroenterology, addiction medicine) [57, 122]. **Interprofessional videoconference for patients with complex needs:** The *Telemedicine IMPACT Plus* program offers patients with multiple chronic diseases and their family physician a one-time videoconference with an interprofessional team (e.g., psychiatrist. Dietician, pharmacist, geriatrician, internist, social worker). The team helps coordinate care planning and identify new solutions for addressing the patient’s needs. A dedicated nurse coordinates the videoconference consultation and provides support to implement recommendations resulting from the consultation [131].	
**13. Drop-in services**	Services are offered to patients who drop-in, without an appointment.	**Drop-in services for youth:** At the *Backdoor Clinic* located in a youth center*,* counsellors and a family doctor provide young people with general health and medical services–sexual health, nutrition, mental health–on a drop-in basis [135].	
**14. Transportation services**	Arranging transportation for patients facing barriers getting to primary healthcare settings.	**Community-based screening program for immigrants:** A screening program to detect unmet health needs among African refugees is held twice a week by a nurse in an apartment complex, where refugees can drop in without an appointment to be evaluated. A translator is available on-site, and a van can transport refugees to a local clinic as needed [82].	
**15. Role expansion or task shifting**	Upskilling of a healthcare worker who has ongoing contact with vulnerable patients to enhance workforce capabilities. May be expansion of the scope of practice for formal providers or training of laypersons.	**Community paramedics for high-risk patients:** Paramedics provide primary care services (e.g., oral and intravenous medication administration, wound care, routine urinary and blood samples, vital sign monitoring) to patients with chronic health concerns who have difficulty getting to their primary care provider or who have low social support [121]. **Nurse-led clinic in disadvantaged neighbourhood:** A cooperative clinic is led by a nurse practitioner specialized in primary healthcare. Through collaboration with nurses, volunteers, psychosocial counsellors and a social worker, the clinic provides comprehensive primary healthcare services to patients living with Hepatitis C or HIV/AIDS and to patients who live in the low-income neighbourhood near the clinic and face multiple barriers accessing the health system [132]. **Interpreter-navigators for refugees:** At an international health clinic that offers primary healthcare to refugees, in-person translators are favoured over a translation phone line. In-person translators have been trained and developed an expertise in how to navigate the system: in addition to translating, they can explain to patients where to go, who to talk to, what to do before tests, financial eligibility requirements, paperwork, etc. [128].	Acceptability Affordability
**16. One-stop shop**	Multiple health and social services are provided in one location to deliver comprehensive care to meet hard-to-reach vulnerable patients’ complex needs at the point of contact.	**In-reach from specialized services to primary healthcare for complex patients:** A visiting geriatrician offers expertise to support a primary health team (family physician, nurses, pharmacist, dietitian and social worker) and, as needed, provides direct care to patients for seniors at risk of cognitive impairment or falling [95]. **One-stop-shop for lesbian, gay, bisexual and transgender (LGBT) and HIV/AIDS patients:** A primary care clinic caters to the needs of LGBT and HIV-positive communities by offering a wide range of safe and inclusive general practice and sexual health services, including HIV prophylaxis treatment, HIV/AIDs management, contraception, sexually transmitted infection screening, hormone therapy, osteopathy (e.g., injuries due to binding), psychology and speech pathology (e.g., for voice feminization) and referrals to LGBT-friendly specialists [130]. **Community center for people at risk of or experiencing homelessness:** The *Living Room*’s team–community development workers, family doctors, nurses, podiatrist, psychologist, nutritionist, mental health counsellors, etc.–provides a range of free services, including health care, food and material aid, phone and internet, mail services, housing support and referrals, legal support, contraception, locker storage, support groups, mental health, alcohol and other drug counselling, podiatry, optometry, hairdressing, yoga, life skills training and art therapy [136].	Acceptability Appropriateness
**Principal accessibility dimension**: **Affordability**			
***Organizational processes and structures that adapt to the economic capacity of people to spend resources and time to use appropriate services.***			
**17. Defraying costs to patients**	Partially or entirely covering direct or indirect costs of accessing primary healthcare.	**Free and low-cost primary healthcare for patients below the poverty level:** Patients 200% below the poverty level are enrolled in a program that allows them to access free visits, laboratories, x-rays and low-cost medications through a network of volunteer primary care providers and specialists [56].	
**Principal accessibility dimension:** **Appropriateness**			
***Appropriateness denotes the fit between services and clients’ needs, its timeliness, the amount of care spent in assessing health problems and determining the correct treatment, and the technical and interpersonal quality of the services provided***.			
**18. Case management**	A healthcare provider (e.g., nurse, social worker) is assigned to individual patients to assess needs, help create care plans, facilitate access to comprehensive services (including but not limited to primary healthcare), coordinate ongoing care, monitor patients and advocate on their behalf.	**Community case management program for vulnerable chronically ill patients:** Chronically ill patients experiencing confusion with medication or treatment plans, frequent emergency department visits or hospitalizations, poor coping skills, in need of education on their condition, inadequate social support, insufficient financial resources or frequently missed appointments are referred to the program. The program is free-of-charge to patients. An advanced practice registered nurse or social worker is assigned to each patient to assess needs, develop care plans, support communication with providers, assist with medication management, coordinate care, help with transportation, focus on development of coping strategies, promote self-advocacy, connect patients with better social support (e.g., health providers, family members, community resources) and conduct regular home visits and telephone follow-ups. Patients are discharged when identified goals are met, and physiological status is stable but are encouraged to contact the case manager if new needs develop [78]. **Post-incarceration case management:** Within two weeks of their release from prison, patients are approached by a community health worker who has completed a 6-month training to enroll in a primary care-based case management program. The community health worker provides referrals to housing, education, employment support, medical and social service navigation; accompanies patients to pharmacies, social services, and medical or behavioural appointments; and supports patients self-manage through home visits, health education and medication adherence support [54].	

^a^Grouped by principal dimension of organizational accessibility

## Discussion

The integration of the scoping review and environmental scan of innovations along with field-testing resulted in a typology of 18 components of organizational innovations to enhance the accessibility of primary healthcare for vulnerable populations. The typology was based on both published and unpublished innovations. It offers a comprehensive menu of potential components that can help inform the design of innovations and can be combined into complex interventions or added to existing organizational processes to meet the access needs of vulnerable populations. Mapping of the components to the accessibility dimensions of the *Patient-Centred Accessibility Framework* [18] allows health service designers to match appropriate innovations to identified access needs.

The typology offers a categorization of health service delivery arrangements inspired by the Cochrane taxonomy of EPOC [20], but tailored to the needs of service designers and specific to the domain of access for vulnerable populations. The ultimate goal is to improve healthcare equity through pro-vulnerable innovation design. A few components are similar to those found in the EPOC and are not specific to vulnerable populations, including *group visits, expanded hours, advanced access, virtual health services, one-stop-shops,* and *role expansion*. However, they have been demonstrated to be well-suited to address the needs of specific vulnerable populations, although organizations still have to be intentional about a pro-vulnerable focus to achieve healthcare equity. Other components in the typology are specifically designed to address the needs of vulnerable populations and differ from the EPOC taxonomy. *Proactive identification of need*, *proactive appointment making and contact,* and *outreach* pull vulnerable persons into primary healthcare and maintain contact rather than placing the onus on vulnerable populations to perceive their needs and navigate the care-seeking process. Similarly, *community health workers, service brokerage,* and *transportation services* bridge the gap between the health system and vulnerable populations.

Despite the intention to make labels and descriptions as mutually exclusive as possible, there was considerable overlap between some components. For instance, *navigation and information* and *proactive identification of need,* although implemented as stand-alone innovations, are also functions of *community health workers* and *case managers*. Likewise, *culturally adapted services* or *inter-organizational pathways* are functions of *community health workers* or *role expansion* innovations; these can be implemented as stand-alone interventions or added to existing organizational processes to address an accessibility need.

### Using the typology in the design of innovations

This typology is intended to provide “building blocks” that can be combined into complex innovations or added to existing organizational processes to address specific access needs of vulnerable populations. The analysis done by Khanassov et al. [17] emphasizes that interventions are most effective in reducing unmet needs, emergency department visits, and hospitalizations when the intervention components are formally coordinated or integrated with other parts of the health system. Integration and coordination are critical considerations in implementing any of these interventions.

The *Patient-Centered Accessibility Framework* [18] is helpful in both identifying the access needs of the population and the components that are most appropriate to address those needs. For example, a population that frequently visits the emergency department may require an innovation that targets Approachability or Acceptability (e.g., *navigation & information, proactive identification of need, culturally adapted services*). Conversely, if the target population is seeking services but is disengaged from ongoing care, then Appropriateness-related components such as *proactive appointment-making, case management,* or *one-stop shops* may be better suited.

It is not surprising that most of the innovation components pertain to Approachability and Availability & Accommodation. Several studies have highlighted that vulnerable populations often face difficulties perceiving health needs, navigating the health system to find services, finding time to obtain services, making an appointment, and finding transportation to reach services [18, 137–139]. This typology emphasizes the importance of addressing barriers early in the care-seeking trajectory and provides a range of potential solutions to mitigate these barriers.

Although other components addressed Affordability as a secondary dimension, it is surprising that only one component in the typology (*defraying costs to patients)* related principally to Affordability*,* especially given the importance of direct and indirect cost as a barrier to care for vulnerable populations [140–143]. This result is partly due to our focus on identifying organizational innovations that can be implemented by a single organization or sub-system. Macro-level ‘innovations,’ such as universal health insurance and the Affordable Care Act, were excluded from our analysis because they are country-level legislative policies rather than organizational innovations.

### Example of how the typology has been used

As mentioned, the typology was field-tested for relevance and usability in our local partnerships between 2014 and 2018 as they designed and piloted innovations to address local access priorities for vulnerable populations. In the local partnership in Quebec (Canada), a preliminary version of the typology was used to inform discussions about the design of an innovation for patients from disadvantaged neighbourhoods who face barriers connecting with a regular primary care provider. The innovation was a combination of several components: trained volunteer community members (inspired by *community health workers*) reached out to patients from disadvantaged neighbourhoods who were on the provincial centralized waiting list to find a primary care provider (existing *primary healthcare brokerage)* to screen for potential access barriers *(proactive identification of need)*. These volunteers provided support by telephone before and after their first visit with their new primary care provider, including discussing the importance of attending the first visit, offering information about the clinic, giving visit preparation materials, and providing general information about the health system (*navigation & information)*. Stakeholders in the local partnership perceived the typology as a useful tool to expand the menu of innovations and to reflect on how components could be added to existing organizational processes. Furthermore, we used the typology and the *Patient-Centred Access Framework* [18] to describe each local partnership’s innovations. This tool allowed the IMPACT research team, which spanned six sites in Canada and Australia with different contexts, languages, and terminology, to clarify their aims, improve mutual understanding across sites, and compare their innovations.

### Implementing components of an innovation

The selection of components as part of a complex innovation also depends on the resources and implementation control available to innovation designers. The decision to implement components such as *group visits, drop-in services, facilitated appointment making, expanded hours, culturally adapted services, case management,* or *advanced access* are generally within the control of a single primary healthcare organization and can be resourced through the reorganization of existing resources. In contrast, components such as *outreach of primary healthcare services, transportation,* and *navigation & information services* require investment of new resources that may be possible for a single organization with a strong commitment and sufficient resources. Other components, such as *role expansion* and *virtual health services*, may require changes at a higher level since they involve changes beyond the reach of a single organization. For instance, *role expansion* – such as nurses working at the top of their scope of practice or empowering front office staff to refer patients to social workers – may require collaboration with professional associations to modify regulations governing professional practice. Similarly, implementing a *community health worker* may require collaboration with other organizations to set up certified training programs or to secure funding to cover salaries. Similarly, *one-stop shops* or *inter-organizational pathways* are based on collaborations between various organizations outside of primary healthcare and require substantial support and political will from local or regional health authorities for implementation.

### Strengths and limitations

Because we achieved saturation with the peer-reviewed articles from the scoping review and descriptions of innovations from the environmental scan, we are reasonably confident that this typology reflects the most common innovation components to improve access for vulnerable populations. We recognize that we have excluded innovations outside the study period. Yet, as 2000 to 2014 represents a period of renewal and reform for primary healthcare marked by an effervescence of innovations, we believe that we have captured the most common components to improve access to primary care. Since 2014, we have continued to test the comprehensiveness of our typology by informally comparing it to innovations described in more recent peer-reviewed studies (although we did not use the systematic approach applied to the literature of the study period). In subsequent presentations to international audiences at conferences, we heard of additional examples of innovations, such as organizational arrangements between hospitals and farmers’ markets, but were able to locate them within typology components. We have not found any new components emerging from more recent literature or examples of innovations to add to our typology. An additional strength of this study is that from 2014 to 2018, the typology was field-tested for usability and relevance with local partners to design pro-vulnerable innovations. We are therefore reasonably confident that, although a new scan or scoping review would add detail and examples to the typology, it would not fundamentally change the components.

We also recognize that our scoping review data, from which we developed our initial typology components, described innovations in only nine OECD countries (Canada, USA, UK, Australia, Germany, New Zealand, Italy, Israel, and Mexico). We minimized the effect of publication bias by doing an environmental scan to identify unpublished innovations, but this method is susceptible to selection bias. Most responses came from Australia and Canada, and running the survey during the Northern Hemisphere’s summer may have limited responses from Europe and the USA. We also have no way of assessing the response rate or comprehensiveness of responses gathered using the social media approach. Although we did have a small number of respondents from low-income countries in our environmental scan, our approach may have excluded some innovative initiatives in low- and middle-income countries (e.g., community-based insurance plans [144], identification of accredited clinics [145], and subsidized payments for primary healthcare services [146]. Another example is the well-known hub-and-spoke models in India [147], which combine *role expansion* with *inter-organizational pathways* to provide low-cost, high-quality services to underserved populations [148].

A final limitation is that the typology labels reflect available examples and the judgment and language of the analysts. Labels such as *proactive identification of need* are clumsy but they circumscribe a unique set of examples.

## Conclusions

This typology is unique as it presents components of innovations that can be put in place by primary healthcare organizations or other health system stakeholders to improve access to primary healthcare for vulnerable populations. Further research on the effectiveness of combining different components may help inform efforts to improve access for vulnerable populations.

## Supplementary information

**Additional file 1.** Example of Search Strategy for Embase.

## Data Availability

The datasets used and/or analyzed during the current study are available from the corresponding author on reasonable request.

## Abbreviations

EPOC: Effective Practice and Organization of Care
IMPACT: Innovative Models Promoting Access-to-Care Transformation
OECD: Organization for Economic Cooperation and Development
