# Supplementary material for: Typology of organizational innovation components: building blocks to improve access to primary healthcare for vulnerable populations
Source: Int J Equity Health. 2020 Oct 6;19:174. doi: 10.1186/s12939-020-01263-8 (PMC7541234; doi:10.1186/s12939-020-01263-8)
Supplement: Supplementary file 1 — Additional file 1. Example of Search Strategy for Embase. [file 12939_2020_1263_MOESM1_ESM.docx]

**Additional file 1: Example of Search Strategy for Embase**

1 translational research/ (5528)

2 integrated health care system/ (6588)

3 Case Management/ (7314)

4 Knowledge Management/ (762)

5 exp quality control/ (220922)

6 health care quality/ (153298)

7 Organi?ational innovation*.tw. (71)

8 innovat*.ti. (14842)

9 Organi?ational change*.tw. (1807)

10 organi?ational model*.tw. (548)

11 (diffusion adj2 innovation*).tw. (504)

12 Integrated delivery system*.tw. (569)

13 Integrated Health Care System*.tw. (325)

14 Integrated Health* System*.tw. (544)

15 (program or programs or programme or programmes).tw. (468596)

16 medical care team*.tw. (56)

17 interdisciplinary health team*.tw. (11)

18 healthcare team*.tw. (1537)

19 health care team*.tw. (2704)

20 case management.tw. (7055)

21 managed care.tw. (15501)

22 knowledge management.tw. (838)

23 healthcare quality.tw. (1068)

24 health care quality.tw. (1756)

25 quality of health care.tw. (4484)

26 quality of healthcare.tw. (1334)

27 quality management.tw. (5531)

28 quality assurance.tw. (17475)

29 case coordination.tw. (25)

30 (intervention or interventions).tw. (582350)

31 multidisciplinary team*.tw. (12026)

32 or/1-31 (1321868)

33 health care access/ (34233)

34 health care availability/ (7450)

35 Healthcare Disparity/ (5959)

36 exp patient attitude/ (217912)

37 (availab* adj2 (healthcare or health care or health service*)).tw. (1393)

38 (access* adj2 (healthcare or health care or health service*)).tw. (9122)

39 access*.ti. (40638)

40 program* accessibility.tw. (20)

41 program* availability.tw. (62)

42 affordability.tw. (2214)

43 approachability.tw. (103)

44 appropriateness.tw. (13616)

45 or/33-44 (311948)

46 32 and 45 (86444)

47 exp child health care/ (47406)

48 exp community care/ (63087)

49 Mental Health Services/ (31280)

50 exp Primary Health Care/ (86682)

51 General Practice/ (43336)

52 child health service*.tw. (444)

53 infant health service*.tw. (16)

54 community nurs*.tw. (1833)

55 community mental health service*.tw. (588)

56 community health service*.tw. (551)

57 community pharmac* service*.tw. (123)

58 maternal health service*.tw. (246)

59 preventive health service*.tw. (361)

60 (senior center* or senior centre*).tw. (462)

61 (center* for the aged or centre* for the aged).tw. (334)

62 primary care.tw. (76389)

63 primary health care.tw. (11687)

64 primary healthcare.tw. (2676)

65 general practice.tw. (21225)

66 family practice.tw. (3694)

67 family medicine.tw. (6162)

68 or/47-67 (285192)

69 46 and 68 (15001)

70 Vulnerable Population/ (6013)

71 Poverty/ (23599)

72 Unemployment/ (7459)

73 Homelessness/ (5938)

74 chronic disease/ (97466)

75 mental disease/ (118256)

76 exp *mental disease/ (664947)

77 exp *aged/ (16069)

78 frail elderly/ (5142)

79 very elderly/ (9994)

80 Minority Group/ (8274)

81 Disabled Person/ (12980)

82 exp "Drug Use"/ (150974)

83 medically uninsured/ (315)

84 illegal immigrant/ (131)

85 immigrant/ (8277)

86 indigent/ (329)

87 lowest income grouup/ (0)

88 medically underserved/ (262)

89 refugee/ (5352)

90 exp Terminally Ill patient/ (4703)

91 vulnerab*.tw. (76118)

92 poverty.tw. (13629)

93 high risk population*.tw. (8227)

94 high risk patient*.tw. (28251)

95 complex patient*.tw. (1884)

96 complex need*.tw. (851)

97 sensitive population*.tw. (315)

98 disadvantaged.tw. (6913)

99 (underserved or under served).tw. (5638)

100 indigen*.tw. (18928)

101 tribes.tw. (2017)

102 native*.tw. (124764)

103 aboriginal*.tw. (5424)

104 low income.tw. (17600)

105 unemploy*.tw. (10051)

106 underemploy*.tw. (179)

107 homeless*.tw. (5781)

108 (street people or street person*).tw. (12)

109 social* stigma*.tw. (1141)

110 social* isolat*.tw. (4447)

111 inequalit*.tw. (13899)

112 uninsured.tw. (5190)

113 underinsured.tw. (459)

114 uneducated.tw. (402)

115 low educat*.tw. (4366)

116 poorly educated.tw. (260)

117 illitera*.tw. (3098)

118 chronic disease*.tw. (39799)

119 chronic* ill*.tw. (13506)

120 chronic condition*.tw. (10227)

121 aged.tw. (351635)

122 old.tw. (694632)

123 older.tw. (266367)

124 elderly.tw. (162147)

125 frail*.tw. (10361)

126 (senior or seniors).tw. (24681)

127 functional* impair*.tw. (12914)

128 disabled.tw. (13707)

129 disability.tw. (95888)

130 disabilities.tw. (28088)

131 handicapped.tw. (2633)

132 physically challenged.tw. (54)

133 mentally challenged.tw. (89)

134 mental disorder*.tw. (23602)

135 mental* ill*.tw. (21844)

136 psychiatric diagnos*.tw. (6839)

137 drug use*.tw. (42628)

138 drug abuse*.tw. (12151)

139 drug addict*.tw. (6598)

140 drug dependen*.tw. (4120)

141 drug habit*.tw. (69)

142 "substance use".tw. (19197)

143 substance dependen*.tw. (2410)

144 substance addict*.tw. (285)

145 uninsured.tw. (5190)

146 underinsured.tw. (459)

147 terminal* ill*.tw. (4662)

148 minority.tw. (38757)

149 minorities.tw. (6698)

150 immigra*.tw. (19128)

151 foreigner*.tw. (952)

152 refugee*.tw. (4336)

153 or/70-152 (2572418)

154 69 and 153 (7631)

155 limit 154 to yr="2000 -Current" (7118)

156 limit 155 to (english or french) (6891)

157 qualitative research*.mp. (29502)

158 qualitative stud*.mp. (18918)

159 action research.mp. (2607)

160 Participatory Research/ (1916)

161 participatory research.mp. (3107)

162 case stud*.mp. (68678)

163 ethno*.mp. (67315)

164 grounded theory.mp. (6560)

165 phenomeno*.mp. (137337)

166 Narrative/ (2274)

167 narrative*.mp. (16468)

168 biograph*.mp. (4728)

169 Autobiograph*.mp. (3202)

170 documentar*.mp. (1411)

171 qualitative synthes*.mp. (229)

172 active feedback.mp. (106)

173 conversation*.mp. (11040)

174 discourse*.mp. (9173)

175 thematic.mp. (10376)

176 qualitative data.mp. (6953)

177 key informant*.mp. (3676)

178 focus group*.mp. (25043)

179 case report*.mp. (1137960)

180 exp Interview/ (123867)

181 interview*.mp. (240740)

182 exp Observational method/ (1826)

183 observer*.mp. (52777)

184 visual data.mp. (249)

185 (audio adj record*).mp. (2581)

186 Cultural Anthropology/ (32392)

187 experience*.mp. (670612)

188 or/157-187 (2271697)

189 exp clinical study/ (4260567)

190 exp Methodology/ (2712060)

191 randomization/ (54544)

192 Placebos/ (185259)

193 Crossover procedure/ (35159)

194 or/189-193 (5940545)

195 (clinic* adj25 trial*).mp. (966368)

196 random*.mp. (862922)

197 control*.mp. (5237933)

198 (latin adj square).mp. (2318)

199 placebo*.mp. (243987)

200 or/195-199 (5877623)

201 exp Comparative Study/ (666277)

202 comparative stud*.mp. (469566)

203 Validation Study/ (44621)

204 validation stud*.mp. (50401)

205 evaluation research/ (1614)

206 evaluation stud*.mp. (5068)

207 Follow-Up/ (685736)

208 followup.mp. (21000)

209 follow-up.mp. (902021)

210 cross over.mp. (14288)

211 crossover.mp. (50503)

212 prospective*.mp. (548205)

213 volunteer*.mp. (132028)

214 or/201-213 (2089573)

215 singl*.mp. (1081892)

216 doubl*.mp. (392712)

217 trebl*.mp. (253)

218 tripl*.mp. (76252)

219 or/215-218 (1456962)

220 mask*.mp. (51854)

221 blind*.mp. (236161)

222 220 or 221 (284003)

223 219 and 222 (164051)

224 194 or 200 or 214 or 223 (9418632)

225 exp Health Survey/ (140568)

226 Health Care Survey/ (5933)

227 exp Risk/ (1311750)

228 exp Incidence/ (231106)

229 exp Prevalence/ (360569)

230 exp Mortality/ (512165)

231 cohort*.mp. (395294)

232 case-control.mp. (122486)

233 cross sectional.mp. (214289)

234 (health* adj2 survey*).mp. (155311)

235 risk.mp. (1871315)

236 incidence.mp. (563598)

237 prevalence.mp. (512194)

238 mortality.tw. (486678)

239 "case series".mp. (43403)

240 "time series".mp. (24993)

241 "before and after".mp. (171898)

242 prognos*.mp. (514923)

243 predict*.mp. (1017431)

244 course*.mp. (614624)

245 or/225-244 (4558876)

246 (mixed adj5 method*).mp. (9599)

247 multimethod*.mp. (1003)

248 (multiple adj5 method*).mp. (22707)

249 or/246-248 (33153)

250 qualitative.mp. (135948)

251 Qualitative Research/ (23741)

252 quantitative.mp. (442547)

253 250 or 251 (135948)

254 252 and 253 (48559) approache

255 249 or 254 (79725)

256 188 or 224 or 245 or 255 (10841719) methode

257 256 not (letter/ or editorial/) (10370545)

258 257 not (animal not human).sh. (10070426)

259 156 and 258 (6064)

260 limit 259 to embase (4439)
